# Supplementary material for: Fmr1 KO causes delayed rebound spike timing in mediodorsal thalamocortical neurons through regulation of HCN channel activity
Source: bioRxiv. 2025 Feb 3:2025.02.02.636122. Preprint. [Version 1] doi: 10.1101/2025.02.02.636122 (PMC11838482; doi:10.1101/2025.02.02.636122)
Supplement: Supplement 1 [file media-1.pdf]

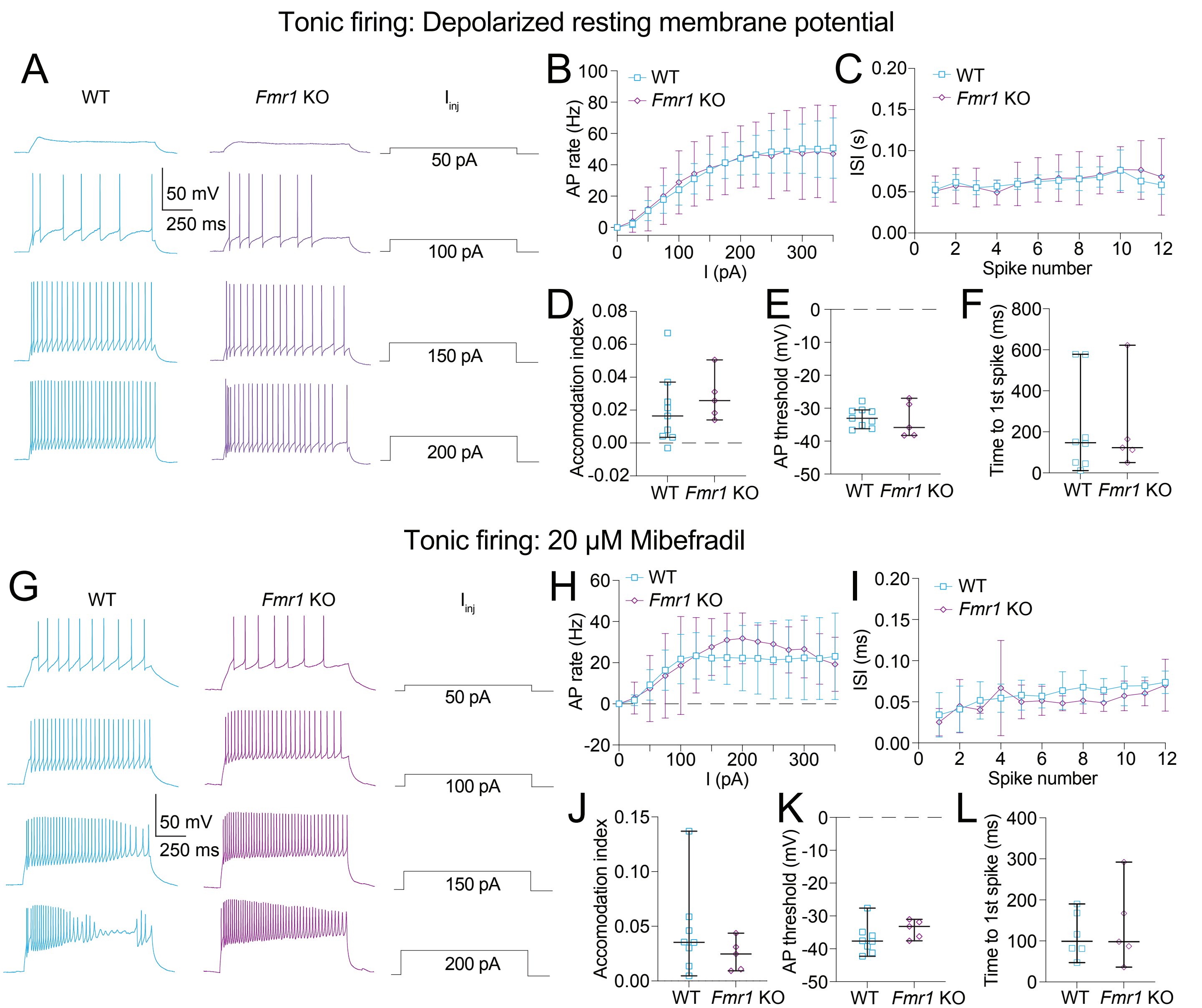

Figure S1: Tonic action potential firing is not different between WT and *Fmr1* KO MD neurons. A-F. Tonic firing data collected from cells that did not fire bursts at resting membrane potential. **A.** Representative traces showing tonic action potential firing in WT and *Fmr1* KO neurons in response to 50, 100, 150, and 200 pA current steps. **B.** Action potential firing rate measured at current steps from 0 to 350 in 25 pA intervals. 2-way ANOVA – effect of genotype: p = 0.97. **C.** ISI of action potentials in the first step with 12 or more action potentials. Fixed effects (type III) – effect of genotype: p = 0.93. **D.** Accomodation index measurements taken from the first current step to elicit 12 or more action potentials. Mann-Whitney: p = 0.3. **E.** Action potential threshold measured from the first step with 12 or more action potentials. Mann-Whitney: p = 0.7. **F.** Time to the peak of the first spike in the first current step to elicit one or more action potentials. Mann-Whitney: p = 0.94. **G-L.** Tonic firing data collected from cells held at -65 mV with the CaV 3 blocker mibefradil added to prevent bursting. **G.** Representative traces showing tonic action potential firing in WT and *Fmr1* KO neurons in response to 50, 100, 150, and 200 pA current steps. **H.** Action potential firing rate measured at current steps from 0 to 350 in 25 pA intervals. 2-way ANOVA – effect of genotype: p = 0.67. **I.** ISI of action potentials in the first step with 12 or more action potentials. Fixed effects (type III) analysis – effect of genotype: p = 0.26. **J.** Accomodation index measurements taken from the first current step to elicit 12 or more action potentials. Mann-Whitney test: p = 0.28. **K.** Action potential threshold measured from the first step with 12 or more action potentials. Mann-Whitney test: p = 0.14. **L.** Time to the peak of the first spike in the first current step to elicit one or more action potentials. Mann-Whitney test: p = 0.93.

Tonic action potential firing is not different between WT and Fmr1 KO MD neurons
To isolate tonic action potential firing, we took two approaches. First, we analyzed that did not fire bursts at RMP (**Fig. S1A-F**). Second, we held cells at -65 mV while blocking Ca_v3 channels with 20 μM mibefradil, which prevents burst firing (**Fig. S1G-L**). We used measures of firing frequency, ISI, spike threshold, and accommodation to assess differences in tonic firing between WT and *Fmr1* KO MD neurons.

No comparison of tonic firing properties at RMP was different between WT and *Fmr1* KO neurons (**Fig S1A-F**). We measured action potential firing rate (**Fig S1B**; 2-way ANOVA – effect of genotype: p = 0.97), ISI (**Fig. S1C**; Fixed effects (type III) – effect of genotype: p = 0.93), accommodation (**Fig. S1D**; Mann-Whitney test: p = 0.3), action potential threshold (**Fig. S1E**; Mann-Whitney test: p = 0.7), and time to first action potential in the first depolarizing step to elicit an action potential (**Fig. S1F**; Mann-Whitney test: p = 0.94) and found no differences.

Similarly, no comparison of tonic firing properties during mibefradil wash-on was different between WT and *Fmr1* KO neurons (**Fig S1G-L**). No difference was identified in measurements of action potential frequency (**Fig S1H**; 2-way ANOVA – effect of genotype: p = 0.67), ISI (**Fig. S1I**; Fixed effects (type III) analysis – effect of genotype: p = 0.26), accommodation (**Fig. S1J**; Mann-Whitney test: p = 0.28), action potential threshold (**Fig. S1K**; Mann-Whitney test: p = 0.14), or the time to first action potential in the first current step to elicit an action potential (**Fig. S1L**; Mann-Whitney test: p = 0.93). These results show that suprathreshold tonic firing is not affected in MD neurons in *Fmr1* KO animals.

Subthreshold properties: MD-M

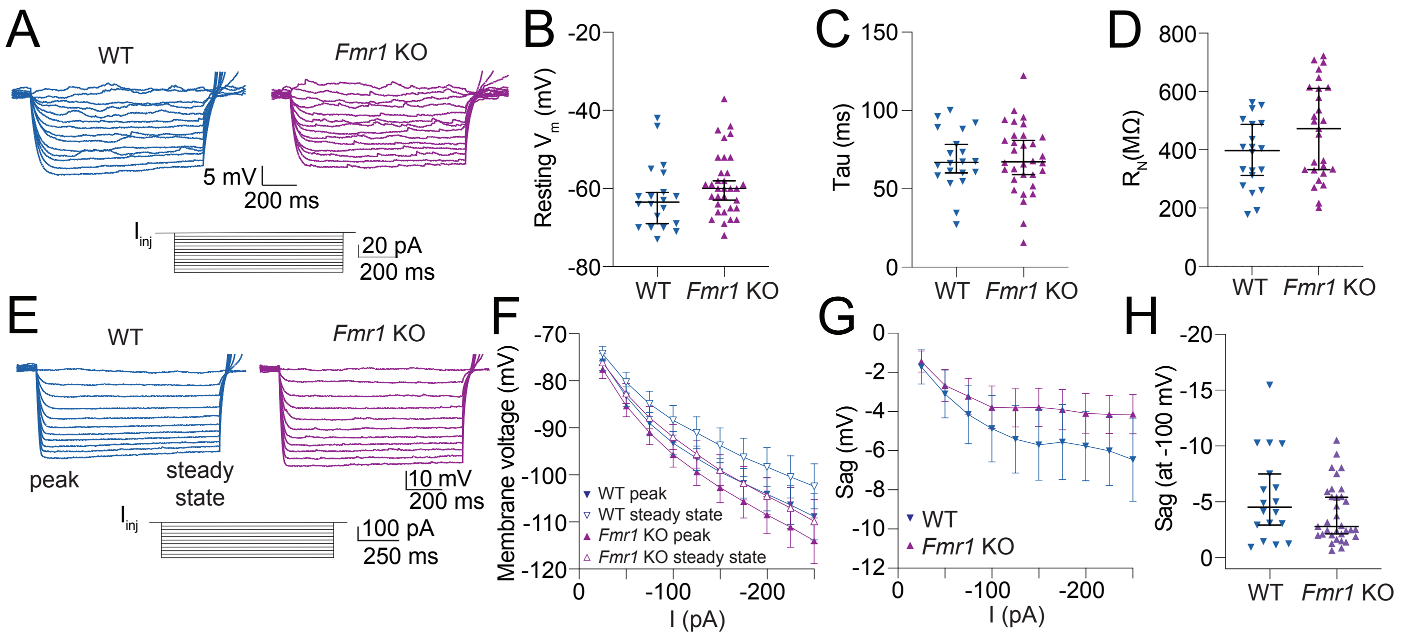

Figure S2: WT and *Fmr1* KO MD-M neurons show no difference in subthreshold properties. A. Representative traces showing voltage responses to hyperpolarizing current steps from 0 to -60 pA in 5 pA intervals in WT and *Fmr1* KO neurons. **B.** Resting membrane potential in WT and *Fmr1* KO neurons. Mann-Whitney test: p = 0.09. **C.** Membrane tau measured from a step to -10 pA in WT and *Fmr1* KO neurons. Mann-Whitney test: p = 0.86. **D.** Input resistance measurements from the linear portion of the IV for WT and *Fmr1* KO MD-M neurons. Mann-Whitney test: p = 0.09. **E.** Representative traces of voltage responses from WT and *Fmr1* KO MD-M neurons to current stimuli ranging from 0 to -250 pA in 25 pA intervals. **F.** VI plot of WT and *Fmr1* KO neurons measured from both the peak and steady state voltage deflections. **G.** Sag, measured as the difference between peak and steady state voltage deflections, in response to current stimuli from -25 to -250 pA in 25 pA intervals. 2-way ANOVA – effect of genotype: p = 0.083. **H.** Sag in WT and *Fmr1* KO neurons when peak hyperpolarization reached -100 mV. Mann-Whitney test: p = 0.24.

Subthreshold properties are not different between WT and Fmr1 KO MD-M neurons

All data discussed so far were from the lateral subnucleus of MD projecting to mPFC (MD-L→mPFC neurons). We tested if these same findings were present in neurons in the medial subnucleus of MD (MD-M→mPFC neurons). We measured voltage responses to current stimuli from -60 to 60 pA in 5 pA intervals (**Fig. S2A**) in WT and *Fmr1* KO MD-M neurons. We found no differences in measurements of resting membrane potential (**Fig. S2B**; Mann-Whitney test: p = 0.09), membrane tau (**Fig. S2C**; Mann-Whitney test: p = 0.86), or R_N (**Fig. S2D**; Mann-Whitney test: p = 0.09). We further investigated the subthreshold property of sag to assess whether changes in HCN activity observed in MD-L were also present in MD-M. We recorded voltage responses ranging from -250 to 0 pA in 25 pA intervals in WT and *Fmr1* KO MD-M neurons (**Fig. S2E**). We created a VI plot from the peak and steady state portions of voltage traces in response to each current step for both WT and *Fmr1* KO MD-M neurons (**Fig. S2F**) to calculate voltage sag at each current step. There was no difference in sag between WT and *Fmr1* KO MD-M neurons either when measured at each current injection amplitude (**Fig. S2G**; 2-way ANOVA – effect of genotype: p = 0.083) or when traces with a peak hyperpolarization of -100 mV were analyzed (**Fig. S2H**; Mann-Whitney test: p = 0.24).

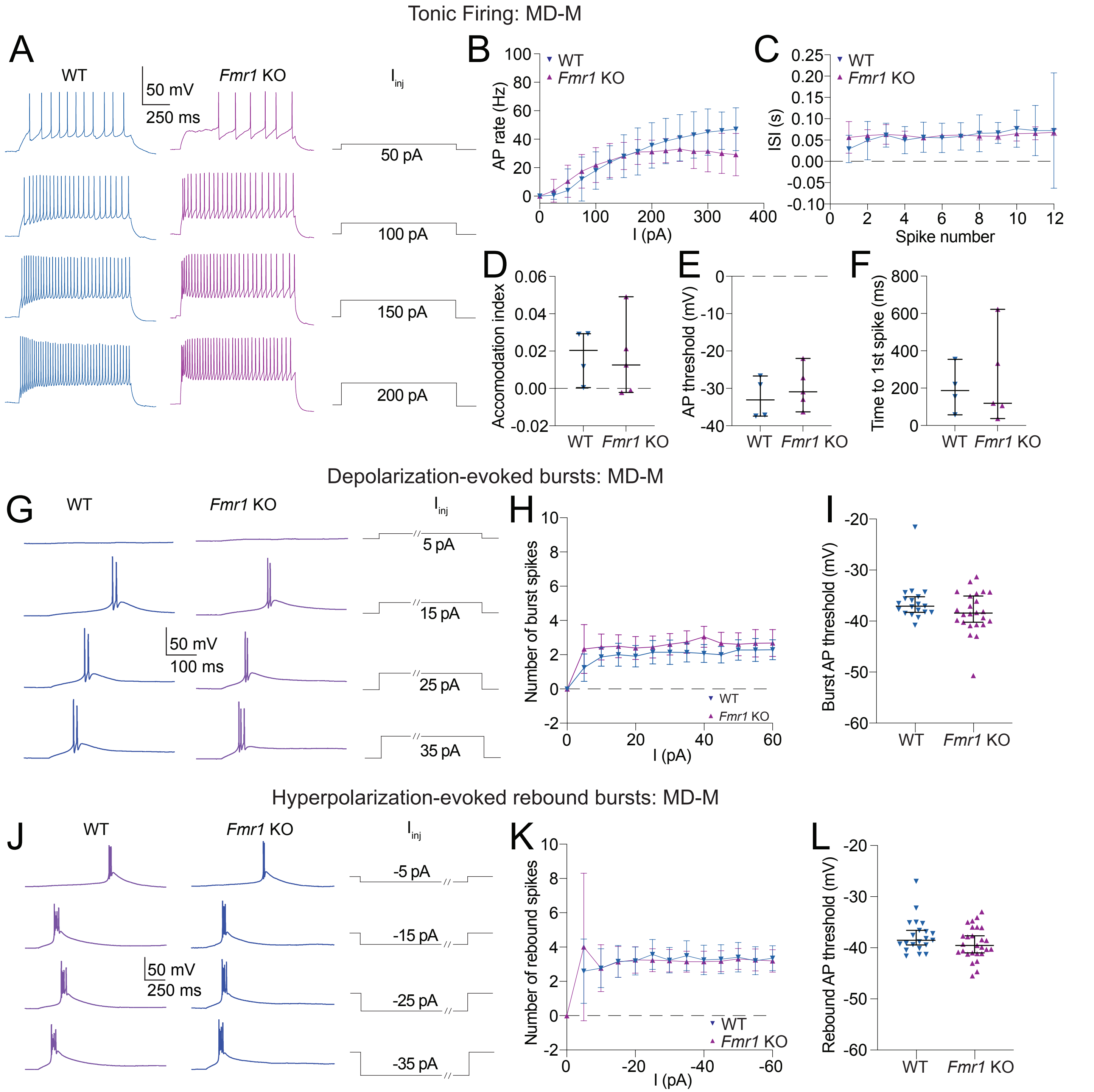

Figure S3: WT and *Fmr1* KO MD-M neurons show no difference in suprathreshold properties. A-F. Tonic firing data collected from cells that did not fire bursts at resting membrane potential. **A.** Representative traces showing tonic action potential firing in WT and *Fmr1* KO MD-M neurons in response to 50, 100, 150, and 200 pA current steps. **B.** Action potential firing rate measured at current steps from 0 to 350 in 25 pA intervals. 2-way ANOVA – effect of genotype: $p = 0.35$. **C.** ISI of action potentials in the first step with 12 or more action potentials. Fixed effects (type III) analysis – effect of genotype: $p = 0.7$. **D.** Accomodation index measurements taken from the first current step to elicit 12 or more action potentials. Mann-Whitney test: $p = 0.73$. **E.** Action potential threshold measured from the first step with 12 or more action potentials. Mann-Whitney test: $p = 0.56$. **F.** Time to the peak of the first spike in the first current step to elicit any action potentials. Mann-Whitney test: $p = 0.9$. **G.** Representative traces showing burst firing in response to 5, 15, 25, and 35 pA steps. Slashes on current steps show where voltage traces were truncated to better visualize bursts. **H.** Number of action potentials per burst for current steps from 0 to 60 pA in 5 pA intervals. Fixed effects (type III) analysis – effect of genotype: $p = 0.32$. **I.** Burst threshold measured from the first step to elicit a burst. Mann-Whitney test: $p = 0.16$. **J.** Representative

traces showing burst firing in response to -5, -15, -25, and -35 pA steps. Slashes on current steps show where voltage traces were truncated to better show bursts. **K.** Number of action potentials per burst for current steps from 0 to -60 pA in 5 pA intervals. Fixed effects (type III) analysis – effect of genotype: $p = 0.9$. **L.** Burst threshold measured from the first hyperpolarizing step to elicit a burst. Mann-Whitney test: $p = 0.18$.

Suprathreshold properties are not different between WT and Fmr1 KO MD-M neurons

We investigated tonic firing from WT and *Fmr1* KO MD-M neurons that did not fire bursts at resting membrane potential (**Fig. S3A**). Our experiments revealed no differences in action potential firing (**Fig. S3B**; 2-way ANOVA – effect of genotype: $p = 0.35$) or ISI (**Fig. S3C**; Fixed effects (type III) analysis – effect of genotype: $p = 0.7$). We found no differences in properties associated with changes in voltage gated channels associated with suprathreshold activity in WT and *Fmr1* KO MD-M neurons including accommodation (**Fig. S3D**; Mann-Whitney test: $p = 0.73$), action potential threshold (**Fig. S3E**; Mann-Whitney test: $p = 0.56$), and time to first action potential in the first step to elicit action potentials (**Fig. S3F**; Mann-Whitney test: $p = 0.9$).

Burst firing was analyzed in depolarizing current steps from 0 to 60 pA in 5 pA intervals (**Fig. S3G**). No difference in either the number of action potentials per burst (**Fig. S3H**; Fixed effects (type III) analysis – effect of genotype: $p = 0.32$) or in the burst action potential threshold (**Fig. S3I**; Mann-Whitney test: $p = 0.16$) was identified between WT and *Fmr1* KO MD-M neurons.

Rebound burst firing was analyzed after offset of hyperpolarizing current steps from -60 to 0 pA in 5 pA intervals (**Fig. S3J**). No difference in either the number of action potentials per burst (**Fig. S3K**; Fixed effects (type III) analysis – effect of genotype: $p = 0.9$) or in the rebound burst action potential threshold (**Fig. S3L**; Mann-Whitney test: $p = 0.18$) was identified between WT and *Fmr1* KO MD-M neurons.

Resource Type	Specific Reagent or Resource	Source or Reference	Identifiers
Add additional rows as needed for each resource type	Include species and sex when applicable.	Include name of manufacturer, company, repository, individual, or research lab.	Include catalog numbers, stock numbers, database IDs or accession numbers, and/or RRIDs. RRIDs are highly encouraged.
Bacterial or Viral Strain	Cholera toxin subunit B (CTB)	Molecular Probes - Thermo Fisher Scientific	Cat# C22841 / RRID:
Chemical Compound or Drug	tetrodotoxin citrate	Abcam	Cat# ab120055 / RRID:
Chemical Compound or Drug	ZD7288	Hello Bio	Cat# HB1152 / RRID:
Organism/Strain	Mouse: C57Bl6	Jackson Laboratory	Stock #000664 / RRID:MGI:3028467
Organism/Strain	Mouse: Fmr1 -/y	Kimberley Huber, Ph.D. (Jackson Laboratory)	Stock#003025 / RRID:IMSR_JAX:003025
Software; Algorithm	Clampex	Axon Intruments	RRID:SCR_011323
Software; Algorithm	Matlab	Wavemetrics	RRID:SCR_001622
Software; Algorithm	Prism	GraphPad	RRID:SCR_002798
Software; Algorithm	Adobe Illustrator	Adobe	RRID:SCR_010279
Chemical Compound or Drug	Mibefradil	Tocris	Cat#2198
